# Optimal Fast Integral Decontamination of *Bacillus thuringiensis* Aerosols and Fast Disinfection of Contaminated Surfaces

**DOI:** 10.3390/microorganisms11041021

**Published:** 2023-04-14

**Authors:** José Luis Pérez-Díaz, Tania Martín-Pérez, Cristina del Álamo, Juan Sánchez-García-Casarrubios, José Luis Copa-Patiño, Juan Soliveri, José M. Orellana-Muriana, Jorge Pérez-Serrano, Francisco José Llerena-Aguilar

**Affiliations:** 1 Escuela Politécnica Superior, Universidad de Alcalá, 28801 Alcalá de Henares, Spain; cristina.alamo@uah.es (C.d.Á.); llerena_fran88@hotmail.com (F.J.L.-A.); 2 Department of Biomedicine and Biotechnology, Universidad de Alcalá, 28801 Alcalá de Henares, Spain; tania.martinp@edu.uah.es (T.M.-P.); josel.copa@uah.es (J.L.C.-P.); juan.soliveri@uah.es (J.S.); jorge.perez@uah.es (J.P.-S.); 3 Counterfog S.L., 28341 Valdemoro, Spain; 4 San Jorge Tecnológicas S.L., 28341 Valdemoro, Spain; jsanchez@sanjorgetecnologicas.com; 5 Animal Experimentation Center, Universidad de Alcalá, 28801 Alcalá de Henares, Spain; jose.orellana@uah.es

**Keywords:** infection prevention, infection control, anthrax, *Bacillus thuringiensis*, airborne pathogens, air decontamination, CBRN, surface disinfection, outbreak management

## Abstract

Aerosolized anthrax (*Bacillus anthracis*) spores are of extreme health concern and can remain airborne for hours and contaminate all kinds of surfaces, constituting reservoirs from which resuspension is easily produced. The assessment of decontamination techniques must therefore consider both air and surfaces. In the present study, several kinds of disinfecting fogs were experimentally tested against *Bacillus thuringiensis* spores, which served as a surrogate for *Bacillus anthracis*, both as aerosols released into the air and spread on porous and non-porous surfaces with different positions and orientations. This technology removed *Bacillus thuringiensis* spores from the air in 20 min with just a 1 min application of fog. The dynamics and characteristics of the fog, related to aerosol and surface interactions, proved to be critical for optimal performance and decontamination. An optimal configuration could provide effective disinfection even on indirectly reached surfaces. In all cases, 8% hydrogen peroxide (H_2_O_2_) provided a higher disinfection rate than 2% glutaraldehyde.

## 1. Introduction

In recent years, the concern about terrorist attacks has increased, reinforcing the necessity of being prepared for these attacks, whether chemical, biological, nuclear, or explosive. Since the biological attacks of 2001 in the United States, where a total of 22 persons were infected and 5 died [1,2], several studies have been conducted related to the mechanisms that allow the early identification of the agent, decontamination methods, and decision-making frameworks that guide sequential actions after a terrorist attack [3,4,5]. Several technologies have been developed, and different disinfectants have been used in different application methods to decontaminate biological agents [4,6,7,8,9]. The biological decontamination approaches consist of the application of chemical liquids and vapors that are sporicidal, such as glutaraldehyde, formaldehyde, peracetic acid, hydrogen peroxide, chlorine, phenol, and heavy metals [10]. Some of these disinfectants, namely chlorine dioxide, ethylene oxide, formaldehyde, hydrogen peroxide, and peracetic acid, have been applied in the gaseous state to inactivate *Bacillus* spores [11]. Disinfectants applied as a fog (in gaseous form) present advantages over liquids, which require wiping and spraying for large-scale decontamination, as fogs are easily dispersed, penetrating unreachable surfaces [9,12]. However, the concentration of the active ingredient in the vapor is necessarily much lower than that in common liquid biocides. Fogging with several disinfectants has been used to decontaminate laboratory and medical equipment, hospitals, healthcare rooms, and animal holding rooms [13,14,15]. Recently, these technologies have been tested on anthrax spores and its surrogates; the preferred disinfectants used were hydrogen peroxide, chlorine dioxide, and peracetic acid [9,16,17,18,19]. Despite the commonly used rationale, claiming that fog reaches the entire volume in a room, the movement of fog droplets is limited by Stokes law and air viscosity. Micron-sized droplets follow the air as it moves, so fog should be considered a fluid made of air plus droplets. Classical disinfectant foggers need a relatively long time for actuation after filling a room. During this time, droplets evaporate, and the biocide acts, eventually, in the gaseous state. In summary, fogging has traditionally been used as a means to achieve the fast vaporization of a liquid biocide.

Additionally, biological decontamination has classically focused on surfaces, forgetting air as a reservoir of pathogens. However, the inhalation of anthrax spores from the air has a much higher mortality rate than that of cutaneous anthrax [20,21]. Only a few technologies consider air sanitation or study effectiveness [22]. Furthermore, most studies have only been conducted against vegetative forms instead of spores [22,23,24].

*Bacillus thuringiensis* has been extensively studied as a suitable surrogate for *Bacillus anthracis* as it also forms small heat-resistant spores [25,26]. Many physical features of these two species are similar. Because size is the most important parameter predicting particle behavior, having similarly sized surrogates is of primary importance [26]. Regarding the comparative resistance and survivability of these two species, more research is needed, but several studies have pointed to *B. thuringiensis* having similar behavior [25] in thermal resistance studies [27] and when the effect of chlorine on these strains was examined [28].

Fog characteristics and properties may vary within a broad range, critically conditioning physical and chemical processes [29]. An open question, therefore, is how fogging procedures and fog properties affect the survival of *Bacillus* spores, either if they are stuck to a surface or if they are airborne.

In the present study, the air and surface disinfection efficacies achieved with the novel Counterfog^®^ fast decontamination system, which generates dynamic fog with different characteristics and biocides, were assessed.

Counterfog^®^ was developed as a rapid response system for collapsing all kinds of dispersed agents by using a nozzle that creates a dynamic jet of fog composed of nanometric liquid droplets. It effectively removes chemical surrogates, nuclear surrogates, and even diesel particles from the air due to their particular dynamics, making particles aggregate and collapse [30,31,32]. Moreover, Counterfog^®^ devices were recently demonstrated to remove bacteriophage Φ29 and SARS-CoV2 virus aerosols from the air in rooms and railway cars [33]. Preliminary tests with *Bacillus thuringiensis* var. kurstaki spores were also performed with encouraging results [34]. This device can produce different types of fog composed of any liquid and dynamics depending on their operating parameters. For surface disinfection, Counterfog^®^ can rapidly disinfect stainless steel surfaces contaminated with bacteriophage Φ29 [35].

In this study, we assessed the effectiveness of three kinds of water fog generated with Counterfog^®^ to effectively remove bioaerosols from the air; additionally, we determined the effectiveness of fog produced with two kinds of biocides to disinfect dried spores on porous and non-porous materials. The main characteristic of the three compared fogs was the droplet size distribution. As a reference, the size of a *Bacillus* spore is approximately 0.8 µm.

The effectiveness of this new technology, when applied with 8% hydrogen peroxide (H_2_O_2_) and 2% glutaraldehyde disinfectants to inactivate bacterial spores on surfaces, was evaluated in a room decontamination scenario. The experimental variables included the positions and localizations of the surfaces, sporicidal liquid, and material.

## 2. Materials and Methods

### 2.1. Media Selection for Spore Production

The microorganism used to carry out the tests was *Bacillus thuringiensis* var. kurstaki (Berliner, 1915), obtained from the Spanish Type Culture Collection (CECT) 4454. It was used as a surrogate for *Bacillus anthracis* endospores due to its genetic similarity and spore size [25,36]. 

A culture medium, 1× Nutrient Broth (NB) (Scharlau, 02-144-500), supplemented with 1% MnSO_4_·H_2_O (according to CECT), was inoculated with a standardized inoculum of *B. thuringiensis* and grown for 1 week at 32 °C in an orbital shaker set at ca. 150–200 rpm. The production of spores was observed at different times with a light microscope (MOTIC, BA-300). To determine viable spore titers, samples of cultures were incubated at 85 °C for 1 h to eliminate vegetative forms; then, serial dilution (1/10) was performed with 1× phosphate-buffered saline (PBS) (Sigma-Aldrich, P5493, St. Louis, MO, USA) and standardized aliquots were placed onto plate count agar (PCA) (Scharlau, 01-161-500, Barcelona, Spain). Inoculated plates were incubated at 32 °C for 24 h, and then colony-forming units (CFU) were counted with an Automatic Colony Counter Scan^®^ 1200 (Interscience, 4370000, Saint-Nom-la-Bretèche, France).

For the surface disinfection tests, the sporulation media were inoculated with a standardized amount of *B. thurigiensis* and grown for 1 week at 32 °C on an orbital shaker set at ca. 150–200 rpm. This culture was incubated at 85 °C for 1 h to eliminate vegetative forms and was centrifuged at approx. 9000–10,000× *g* for 30 min at 2–8 °C. The supernatant was removed, and the pellet was washed twice and resuspended in 12 mL of 1× PBS (Sigma-Aldrich, P5493), diluted to approximately 1 × 10^8^ CFU/mL, and stored at 2–8 °C until use (no longer than 48 h).

### 2.2. Spore Preparation, Air Dispersion, and Measure of Spore Removal Rate

Spores were suspended in 1× PBS to be dispersed into the air (bioaerosols). Bioaerosols were prepared as follows: *B. thuringiensis* was grown in 500 mL of the production selected medium at 32 °C for a week with shaking (150–200 rev min^−1^). Afterward, the culture was incubated at 85 °C for 1 h and was centrifugated at approx. 9000–10,000× *g* for 30 min at 2–8 °C. The supernatant was removed, and the pellet was washed twice, resuspended in 20 mL of 1× PBS (Sigma-Aldrich, P5493), and set at 0.3 units on the McFarland scale, diluted 10^−2^, which corresponds to 9 × 10^5^ CFU/mL with a densitometer (Grant bio DEN-1, Grant instruments). The inoculum was ready to be dispersed into the air with a gun sprayer; the dry air from a compressor was used at a pressure of 200 kPa. The real CFU per cubic meter (CFU/m^3^) (aerosolized concentration) was determined after spraying the bioaerosol and performing CFU analysis by collecting the air with a particle impactor MAS-100 NT^®^ (Merck, Darmstadt, Germany). Moreover, during the assay, the size (0.3, 0.5, 1, 2.5, 5, and 10 µm of diameter) and total number of particles in the air, temperature, and relative humidity were monitored with an 8306 Handheld Particle Counter (Particles Plus, Stoughton, MA, USA).

**Figure 1:**
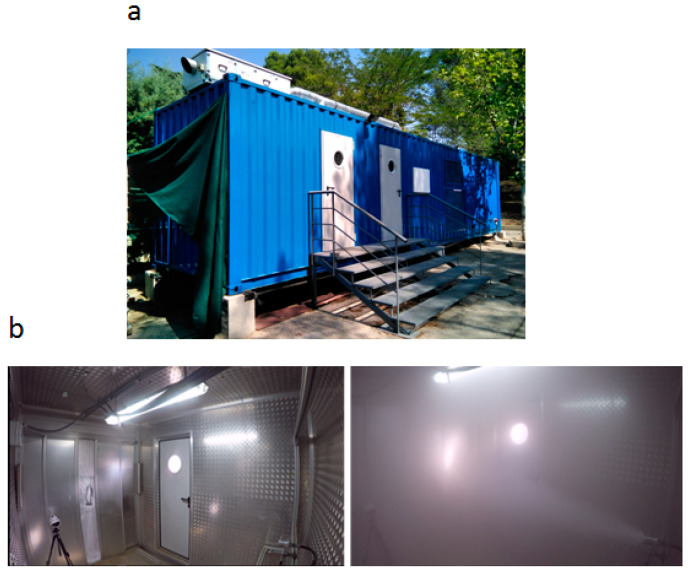
(**a**) Fog Dynamics Laboratory. (**b**) Test room of the Fog Dynamics Laboratory.

Tests were conducted to determine the rate of biological decay removal of *B. thuringiensis* spores. Aerosols were released in the Fog Dynamics Laboratory. The laboratory features were described by Pérez-Díaz et al. (2019) [32]. The laboratory was built in a portable standardized container (12.031 × 2.348 × 2.90 m), which was divided into three rooms: a control room and two test rooms (16.24 m^3^, each test room) (Figure 1). The control room contained all of the different control elements that regulated the temperature and relative humidity in the test rooms, including a heat pump, cooler, and humidifier/dehumidifier. The temperature could be controlled between 0 °C and 40 °C, and the relative humidity between 30% and 95%. The temperature and relative humidity were maintained at 2 °C and 5%, respectively. The Fog Dynamics Laboratory is equipped with a climatization system embedded in the walls, floor, and ceiling, providing maximal homogeneity and smooth thermal conditions in the test room (Figure 1). 

At different times after the dispersion of spores (1, 3, 7, 10, 15, 20, 30, 40, 50, and 60 min), a sample of 50 L (0.05 m^3^) of air was taken by a particle impactor (Merck, MAS-100 NT^®^). PCA plates were incubated at 32 °C for 24 h, and CFU/50 L was counted with an Automatic Colony Counter Scan^®^ 1200 (Interscience Ref.: 437000). This assay was performed in triplicate.

### 2.3. Air Disinfection Using Water Fog 

Aerosol droplets or airborne particles remain floating in the air for hours due to air viscosity [37]. They are dragged by any airflow and only fall down slowly in still air to eventually lay on the ground or surfaces. Additionally, small spores or particles can be easily resuspended by any gentle breeze. Counterfog^®^ was developed to generate tunable water fog for the decontamination of air by removing and collapsing airborne particles. Removing spores from the air, even just to collapse them onto surfaces, additionally preventing resuspension, can be considered sanitization, disinfection, or decontamination. These three terms are usually accepted for logarithmically decreasing pathogen concentration. In this study, we use them interchangeably, but it should be understood that the proper use of the terms implies a particular level of reduction.

Counterfog^®^ is based on a special nozzle [38] that is able to provide a jet of fog, only requiring compressed air and water supply to function. It is able to create a tunable fog, mostly composed of 0.05–20 μm liquid droplets. In the present study, we used three above-described fogs made of water, essentially with decreasing droplet sizes, to disinfect the air. The droplets in the fog were expected to interact with the dispersed spores, aggregating and collapsing them. The collapse of spores and droplets additionally causes droplets to fall. The bigger they become, the faster they descend. Airborne particles can only collapse with liquid droplets of similar size: they cannot coalesce with droplets of a larger size, such as those produced by conventional sprinklers or sprayers, due to the large airflow generated by bigger droplets [39].

Assays were conducted with three different fogs with progressively smaller water droplets. All water fogs were produced in the laboratory room, approximately 750,000 particles/m^3^ of 5 µm water droplets. Fog number 1 additionally provided an almost similar concentration of 10 µm droplets (approximately 700,000 particles/m^3^), whereas fog number 2 provided half this number of 10 µm water droplets. Fogs 1 and 2 provided only 50,000 particles/m^3^ of 2.5 µm-droplets. Finally, fog number 3 provided a much higher number (700,000 particles/m^3^) of small 2.5 µm droplets and considerably fewer (150,000 particles/m^3^) 10 µm droplets (Table 1). 

The experiments were performed in triplicate. In all cases, a spore suspension was released into the air in the test room as an aerosol. Then, 2 min later, a single Counterfog^®^ nozzle in a fixed position was activated for 1 min.

In all the experiments, samples of air were obtained (Table 2), and the CFU/m^3^ of *B. thuringiensis* was measured, as previously described. 

In the experiments, the relative humidity reached levels of 90–100% during the water fog phase, and the temperature was 20 °C.

### 2.4. Test Materials and Coupons Inoculation for Surface Disinfection Tests

The surfaces of buildings and objects (fomites) are known to be reservoirs of microorganisms that can be transmitted either by direct contact or by resuspension. The standardized assessment of biocides consists of spreading a suspension with microorganisms on a stainless steel or glass coupon, application of the biocide, and measurement of the survival rate of the microorganisms. Stainless steel and glass are non-porous surfaces that are adequate for the food industry or operating theaters. However, most buildings and objects include porous materials with more complex surfaces.

A total of 7 materials were used in this study, including porous and non-porous surface materials that can be found in buildings. These materials included: wood laminate from a desk, plasterboard from a wall, a steel metal beam, galvanized steel from a ventilation duct, PVC from a fire extinguisher sign, PVC from a pipe, and a glass microscope slide (deltalab, D100005, Barcelona, Spain).

Each material was cut into 2 × 5 cm pieces (coupons). These coupons were visually inspected before and after decontamination to detect any damage to the materials. 

**Figure 2:**
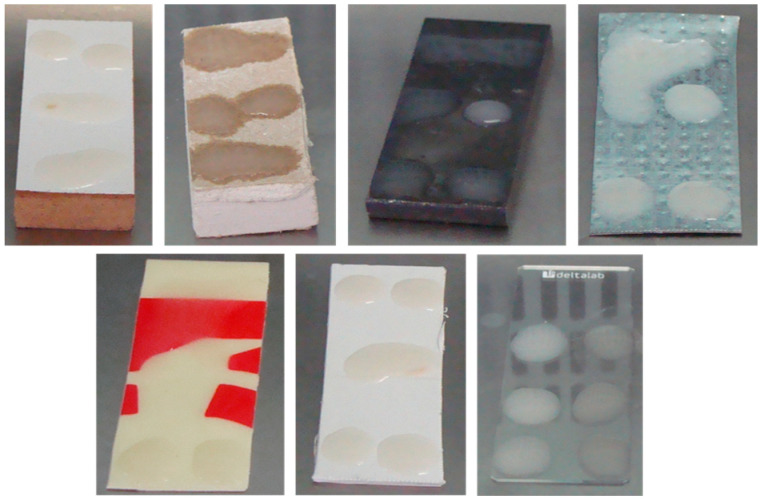
Test coupons inoculated with *B. thuringiensis* spores.

Prior to inoculation with *B. thuringiensis*, the test materials were cleaned by wiping them with 70% ethanol and leaving them in UV light overnight. Each test coupon was horizontally placed and contaminated with a suspension of 1 × 10^8^–4 × 10^8^ spores/mL (Figure 2). The suspension was applied over the surface of the coupons in the form of small droplets (6 droplets of 50 µL each) at a final concentration between 1 × 10^7^ and 4 × 10^7^ spores/coupon. Then, the coupons were dried overnight in a biosafety cabinet. The dry contaminated coupons were placed in sterile and conical 50 mL tubes and stored until the contamination assays were ready. Several coupons for each material type were not inoculated for comparison with coupons that were not contaminated (blank) and other contaminated coupons that were not exposed to the decontamination system (negative controls).

In addition, 0.1 mL of the inoculum was serially diluted in 1× PBS and plated to quantify the inoculum spore titer on the day of coupon inoculation. 

### 2.5. Surface Disinfection Procedure

In these decontamination assays, 8% hydrogen peroxide or 2% glutaraldehyde with phenol (<10%) (Instrunet Esporicida 30, Inibsa Laboratorios, Barcelona, Spain) (hereafter referred to as 2% glutaraldehyde) was used as the biocide. Both disinfectants are certificated according to EN 1650, EN 1276, and EN 13704 standards, reported to be effective against fungi, bacteria, and spores.

**Figure 3:**
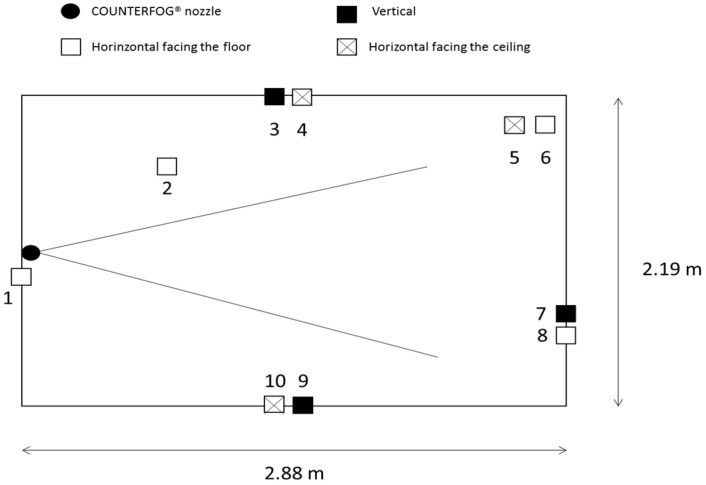
Location and orientation of the plasterboard coupons (top view) in the Fog Dynamics Laboratory in the first group of assays.

The assays were also performed in the Fog Dynamics Laboratory under isothermal conditions. Two kinds of assays were performed with each disinfectant: a test to measure decontamination rates of plasterboard coupons located in different positions in the room and with different orientations—vertical, horizontal upwards, and horizontal downwards (Figure 3). The aim of this test was to reveal the inhomogeneity of the decontamination or how it depends on the relative position with respect to the fog generator. Ten positions were used:

Position 1: horizontal facing downward, just above the nozzle.
Position 2: on the ceiling facing downward, 1 m away from the nozzle.
Position 3: 1 m high, vertical on the middle of the left wall.
Position 4: 1 m high, horizontal on the middle of the left wall.
Position 5: on the floor facing upward.
Position 6: on the floor facing downward.
Position 7: 1 m on the vertical wall, 2.88 m in front of the nozzle.
Position 8: 1 m high, horizontal downward on the wall, 2.88 m in front of the nozzle.
Position 9: 1 m high vertically on the wall with the door.
Position 10: 1 m high, horizontal upward.

The second type of assays were carried out with coupons of 7 different materials to assess the penetrability of each disinfectant’s micro-sized drops created with Counterfog^®^ for the different types of surface materials.

In both assays, the decontamination conditions were as summarized in Table 3: Fog release lasted 1 min, and the test room remained closed until the fog disappeared. After the disappearance of the fog, the ventilation system was activated. After 10 min, the coupons (samples and blank) were placed in a 50 mL conical tube that contained 20 mL of 1× PBS with 200 µg of catalase (Sigma-Aldrich, C1345) for the 8% hydrogen peroxide assays (Rogers et al., 2005) or 0.2 mL of glycine (Sigma-Aldrich, PHG0016) for the 2% glutaraldehyde tests [40]. These were used to neutralize and stop the action of the biocides. The conical tubes were opened in a non-sterile environment. Negative control coupons (contaminated coupons not exposed to fog) were placed in a 50 mL conical tube that contained 20 mL of 1× PBS. Each assay was performed at least twice.

### 2.6. Sample Processing and Quantification of Spore Survival in Surface Disinfection Tests

The conical tubes with the coupons and 1× PBS + neutralizer were shaken at 250 rpm for 30 min to extract the spores from the coupons and then heated at 85 °C to kill the vegetative forms. Then, the samples were serially diluted (1/10), placed (100 µL) onto PCA plates, incubated at 32 °C overnight, and the CFU per milliliter (CFU/mL) was quantified.

### 2.7. Sanitation and Disinfection Efficacy Calculations and Statistical Analysis

To calculate the efficacy of air sanitation, the numbers of viable spores counted at different times were compared with the number of present viable spores immediately after bioaerosol release (sample *t*_1_). The log_10_ reduction was calculated using the following equation:

(1) $$\mathrm{Log}\;\mathrm{Reduction}\;={\;\mathrm{Log}}_{10}\;\left(t_{1}/t_{x}\right)$$

where *t*_1_ is the number of viable organisms recovered 1 min after the dispersion of spores, and *t_x_* is the mean number of viable organisms recovered from the samples at different times.

Additionally, the reduction percentage was calculated for each of the samples by comparing the number of colonies in the first sample with the number of colonies in the rest of the samples, according to the following equation:

(2) $$P=100\times \left(t_{1}-t_{x}\right)/t_{1}$$

Efficacy calculations were performed according to Rogers et al. [16]. The log10 reduction was calculated via:

(3) $$\mathrm{Log}\;\mathrm{Red}={\mathrm{Log}}_{10}\left(N/N^{\prime }\right)$$

where *N* is the mean number of viable organisms recovered from the control coupons (negative control), and *N*′ is the mean number of viable organisms recovered from coupons after decontamination (sample coupons).

The reduction percentage was calculated for each of the samples by comparing the number of colonies recovered from the control coupons with the number of colonies recovered from the sample coupons, according to the following equation:

(4) $$P=100\times \left(N-N^{\prime }\right)/N$$

Moreover, the percent recovery was calculated for each type of test material inoculated with spores, as explained by Rogers et al. [16].

Statistical comparisons between the different assays were performed with *t*-tests with no adjustment for multiple comparisons and one-way ANOVA using GraphPad Prism version 5 software, considering *p* < 0.05 as a statistically significant difference.

## 3. Results

### 3.1. Aerosolization

**Figure 4:**
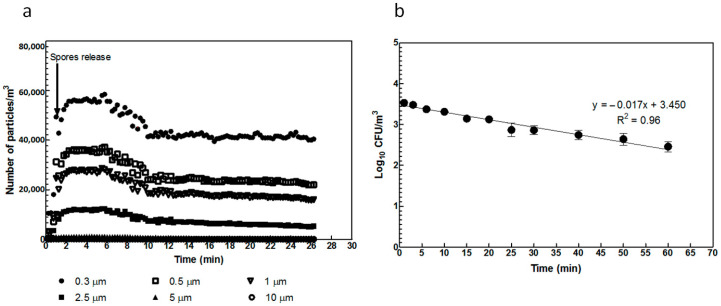
(**a**) Time course of number and size of particles when *B. thuringiensis* spore suspension was released as aerosol in the test room. (**b**) *B. thuringiensis* spores recovered from air with an impactor after dispersion as aerosols. Spore number data are expressed on a logarithmic scale. The vertical lines represent the standard deviation of the media. A least square linear regression is also presented.

Figure 4a shows the time course of the number and size of airborne particles measured with optical techniques after the release of a bioaerosol containing *B. thuringiensis* spores. Figure 4b shows the time course of the number of CFU per cubic meter recovered by impaction and cultivation. The concentrations of particles (0.3, 0.5, 1, and 2.5 µm) suddenly increased after the spore suspension was aerosolized, then they presented a first period of stability of approximately 4 min and a relatively quick decay also of approximately 4 min. Later, they slowly and smoothly fell. The first period could be associated with a relative remaining turbulent flow in the laboratory due to inertia that resuspends all volatile matter, maintaining an almost constant concentration of airborne particles for all sizes. The last period corresponded to an essentially still-air condition where particles slowly fell down (the smaller, the slower). The quick decay between the periods was a transition time in which evaporation and interaction with walls became relevant.

The falling rate (1-log_10_/hour) of *B. thuringiensis* aerosolized spores shown in Figure 4b agrees with that of droplets that are slightly bigger than 1 µm droplets in still-air conditions. The concentration of recovered spores just after aerosolization (3.56 × 10^3^ ± 1.47 × 10^3^ CFU/m^3^) was 7.8 times lower than that of 1 µm droplets. Note that the optical particle counter could not distinguish droplets from particles and only droplets bigger than 1 µm can host a 0.8 µm spore. These figures are, therefore, consistent and imply that the generated aerosol behaved as the finest possible dispersion of Bacillus spores. 

### 3.2. Air Disinfection 

**Figure 5:**
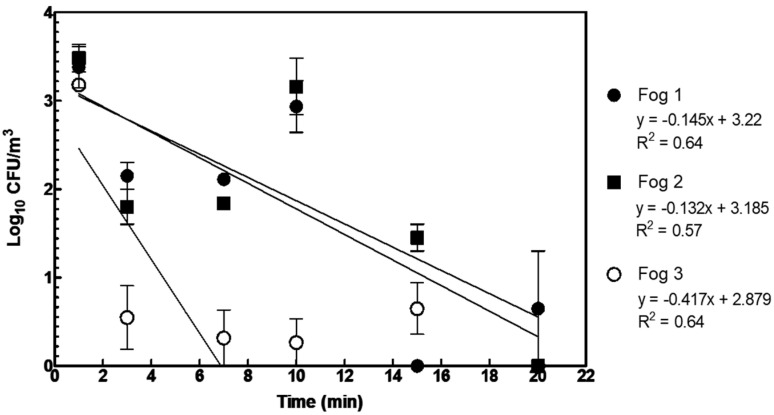
Log10 CFU/m^3^ obtained after fog 1, fog 2, and fog 3 release. In the cases where no CFU was found, it is represented as 0 instead of minus infinite. Linear regressions are also presented.

The jet of fog created by Counterfog^®^ has the physical effect of removing particulate matter, including smoke, from the air [32]. Therefore, for the first decontamination of air, potable water could be used as the liquid for the generation of the decontaminating jets of fog. Aerosolized spores or droplets carrying spores would presumably aggregate and collapse onto the surfaces onto which the jet was aimed. These spores would not be killed: they would be just removed from the air. Tests to experimentally measure the effect of the three kinds of water fog in the removal of bioaerosols released were conducted (Figure 5). First, this was useful for studying the purely physical removal of aerosolized spores that could still be extrapolated to fogs generated with other liquids, including biocides. Second, fast decontamination of aerosols (preventing inhalation) using water fog alone, with absolutely no toxicity and, therefore, no secondary effect, as tested, may have relevant practical applications.

After 1 min of fog production (fog 1), the maximum achieved reduction (3-log10) was observed for sample *t*_5_ (13 min after fog release). When fog 2 was used, a 99.07% reduction (2.03-log10) was obtained for sample *t*_5_ (13 min after fog production). Finally, a 99.16% reduction (2.08-log10) was obtained when fog 3 was produced (sample *t*_3_, 5 min after fog production) (Table 4). Additionally, comparing the natural removal and decontamination rate of the three fogs, statistically significant differences were observed in fog 1 (*p* = 0.012), fog 2 (*p* = 0.015), and fog 3 (*p* < 0.0001). Fog 3, which had a larger number of the finest 2.5 µm droplets, had a higher removal rate of spores (the removal slope was 10 times higher than for the other two fogs composed of bigger droplets). 

### 3.3. Surface Disinfection Using Counterfog^®^ System 

After the decontamination tests, the coupons exposed to disinfectant fogs were observed, and no visible surface damage was present in the material.

The percent recovery of the viable microorganisms in the coupons varied according to the material (Table 5). This recovery was higher from non-porous materials such as PVC from a fire extinguisher sign, PVC from a pipe, and glass. Other materials that are considered non-porous (steel metal and galvanized steel) had a percentage recovery similar to that of porous coupons (wood and plasterboard). The steel metal beam coupon had a rough surface, and the surface of the galvanized steel coupon was pitted. These factors could have influenced the adhesion of the spores to the surfaces as well as their survival and recovery.

In these assays, only the plasterboard coupons were the most difficult to decontaminate porous material. The Counterfog^®^ jet bounced and flowed around the room, presumably colliding with or impacting the walls, ceiling, and floor. In these tests, the reduction in the number of spores with respect to those inoculated on the coupons was measured depending on the position and orientation within the Fog Dynamics Laboratory (Table 6). We found no statistically significant differences (*p* = 0.68) in the log_10_ reduction according to the position or orientation of the coupon. In this assay, the hydrogen peroxide fogs created with Counterfog^®^ were more effective than the glutaraldehyde fogs (*p* = 0.0021). No viable organisms were detected in any of the blank coupons, demonstrating that no transport of viable spores occurred due to the action of the jet of fog.

The 8% hydrogen peroxide fogs created by Counterfog^®^ reduced the number of spores in porous and non-porous coupons with a log_10_ reduction ranging from 1.7 to 4.2 (Table 7). A lower log_10_ reduction (*p* = 0.0002) ranging from 0.2 to 2 was observed in all the coupons when treated with 2% glutaraldehyde fogs using Counterfog^®^ (Table 7). Decontamination was more effective for porous than for non-porous coupons in all the decontamination cases. Among the non-porous coupons, a higher reduction was achieved for the glass coupons. Again, no viable organisms were detected on any of the blank coupons.

A conventional fogging device (Binomiun NouvAIR) was used for comparison with Counterfog^®^ technology with a commercially available system. The observed log_10_ reduction in viable spores inoculated on all the coupons tested ranged from 0.29 to 2.05 when H_2_O_2_ fogs were created with this conventional (non-dynamic) fogging device (Table 8). The differences observed between this device and Counterfog^®^ were statistically significant (*p* = 0.0009).

## 4. Discussion

The small (0.8 µm) size of anthrax spores makes them extremely volatile and able to remain airborne for many hours. Additionally, they are extremely resistant to extreme environmental conditions, which makes them a preferred bioweapon. In the case of the contamination of any facility, integral decontamination of materials, devices, furniture, and air will be required. Ideally, decontamination technology should be fast and simultaneously respectful of materials, particularly electronics and electrical devices. Counterfog^®^ dynamics technology was developed for that purpose [41]. It provides a jet of fog composed of nanometric droplets with two simultaneous actions: capturing airborne particles and droplets and projection onto surfaces, creating an extremely thin film (from tens of nanometers to a few microns thick) that lasts a few minutes before evaporating. The amount of liquid and time to evaporate are critical: the amount must be large enough to complete the disinfection of the microorganism burden but simultaneously be small enough to minimize damage to materials as well as to any electronics. 

The first decontaminating action is based on physics, particularly interface physics. Therefore, a demonstration with water can be extrapolated to any water-based liquid (for example, a hydrogen peroxide solution). The second action of creating a thin liquid film can be used for the decontamination of surfaces as well as of the matter collapsed from the air onto the surfaces, provided the liquid has a biocidal effect.

In this paper, we present the results of experimental tests assessing the ability of Counterfog^®^ dynamics technology to integrally decontaminate *Bacillus thuringiensis* var. kurstaki spores from both surfaces and air in a 16.24 m^3^ room. *B. thuringiensis* was used as a surrogate for *B. anthracis*. The genetics, size, and composition of the *B. thuringiensis* spores are similar to those of *B. anthracis* [25,36,42]. Sagripanti et al. 2007 showed that *B. thuringiensis* and *B. anthracis* spore inactivation are similar when treated with products containing peroxide, chlorine, or oxidants [10]. Decontamination with hot, humid air also resulted in the similar inactivation of *B. thuringiensis* and *B. anthracis* spores [8]. As such, decontamination tests can be conducted with surrogates that can be extrapolated to *B. anthracis*.

Two kinds of disinfection tests were performed: dry aerosols and surfaces.

Our results showed that the dry aerosolization of spores was efficient, with a slow particle falling rate of 1-log_10_/h, as shown in Figure 4b. This agrees with the dynamics of droplets that are slightly bigger than 1 µm in still air. 

The spore concentration recovered just after aerosolization (3.56 × 10^3^ ± 1.47 × 10^3^ CFU/m^3^) was 7.8 times lower than the concentration of 1 µm droplets. Note that the optical particle counter could not distinguish droplets from particles, and only droplets bigger than 1 µm could host a 0.8 µm spore. These figures are, therefore, consistent and imply that the generated aerosol behaved as the finest possible dispersion of Bacillus spores.

Additionally, the concentration of spores could be controlled to be able to quantify when the different methods of decontamination were used. Our data indicated that we were able to create systems in which the spores remain in the air for more than 60 min. Unlike other air sanitation methods, no muffin fan or other devices were used to distribute the microorganisms in the air. Similarly, other researchers simply studied the microorganisms that are naturally present in the air [23,24,43]. The results reported in this paper show that Counterfog^®^ reduces the spores in the air by at least 3-log_10_ when a nanometric fog just composed of water is applied for 1 min. Fog 3, with a larger number of the finest 2.5 µm droplets, produced an increased removal rate of spores compared with the other two fogs studied (which were composed of larger droplets). We, therefore, found that size and dynamics are key. Only droplets with a size similar to that of the aerosol particles collapsed quickly and efficiently. A 3-log_10_ reduction in the microorganisms on surfaces or in the air is the recommended value established by the U.S. Environmental Protection Agency (EPA, 2012) [44]. This was achieved by Counterfog^®^. This figure may be higher, but to determine if this is possible, a higher concentration of spores released should be used. However, as stated by Sattar et al. (2017) and confirmed by us, a larger number of CFUs in the air in control samples limits the accurate detection of colonies and count [24].

Air cleaning technologies based on HEPA filtration provide a 3-log_10_ reduction in 90 min [24], and devices based on UV light and high-efficiency particulate arrestor filtration achieve these levels in 38–45 min [22]. In contrast, Counterfog^®^ presents several advantages over those technologies: (a) a 3-log_10_ reduction is reached in only 20 min (with a 1 min application), being faster than the other known devices; (b) a substantially lower cost than the filtration method; (c) isolation of the area (room, building, etc.) is not needed for the cleaning process; and (d) toxic disinfectants are not necessary. If no disinfectant is used, the spores remain viable on the surface where the fog is projected, so a second step must be performed to kill the viable spores on surfaces. The advantage of this two-step procedure for decontamination is that the first step can be performed even if people have not been evacuated from a contaminated site, as water is non-toxic, preventing people from breathing in the spores.

As mentioned above, decontamination by fogging has various advantages in comparison with other decontamination methods [9,12]. A number of conventional devices gently release fog composed of biocides, promoting their vaporization. These technologies have been widely used in healthcare environments [45,46,47]. Recently, the effectiveness of these fogging devices to decontaminate *B. anthracis* and surrogate spores was assessed, showing the inactivation of the spores [10,17,19]. Counterfog^®^ provides an advancement by generating dynamic fog cones with several advantages over commercially available fogging devices. First, this system expels a cone of pressurized mist that rapidly fills a room. Typically, a 16 m^3^ room can be filled in 30 s [41]. Compared with conventional foggers, this is between 10 and 30 times shorter. In addition, this fog cone reaches a distance of 2 m from the application nozzle, interacting with the local airborne matter, demonstrating that it is an appropriate technology for large-scale decontamination [41]. Another advantage is the capability to create fogs with any kind of liquid or disinfectant, almost independent of surface tension and viscosity. Moreover, the size of the disinfectant drop can be controlled; therefore, the time they remain suspended in the air can be controlled as well. Finally, the contact of the disinfectant with the contaminated surfaces can be narrowly controlled.

The method of coupons used in this study to assess surface disinfection measured up to a 6 log_10_ reduction [19]. The recovery was lower from porous materials than from non-porous materials, as previously reported in other studies [16,48,49].

The observed log_10_ reduction in viable spores inoculated on all the coupons tested ranged from 1.74 to 4.17 when H_2_O_2_ fogs were created with Counterfog^®^ and only from 0.29 to 2.05 when H_2_O_2_ fogs were created with a conventional (non-dynamic) fogging device. Counterfog^®^ is, therefore, more effective than this conventional fogging device.

Notably, the log_10_ reduction was higher for porous than for non-porous surfaces. Other studies using other methods have reported the opposite results [16,17]. This means that Counterfog^®^ is more effective (and faster) at providing H_2_O_2_ droplets and having them absorbed by the surface of materials. In the case of porous materials, it seems from the presented data that the disinfectant was retained in the material and, thus, the decontamination was more effective.

Compared with that used in previous studies [12,16,17,19], the concentration of H_2_O_2_ used in this study is low. In this study, we used 8% H_2_O_2_; previous researchers used 8–35% H_2_O_2_ for a longer exposure time (between 20 min and 168 h). Concentration and time of exposure are the two factors that influence the decontamination rate: to achieve higher log_10_ reduction, these two factors can be increased. 

The reduction in the spores using decontamination with 2% glutaraldehyde fog was lower than that achieved with H_2_O_2_ fog, which could be because glutaraldehyde is less volatile, so more glutaraldehyde must be added to reach a given concentration [50].

Counterfog^®^ can be used as a first-responder decontamination phase, which was established in the decision-making framework developed by Raber et al. [5]. The aims of the first-responder phase are the stabilizing and isolating of the CBRN incident. In this phase, materials or methodologies that are hazardous (known as HAZMAT) are normally used [5]. However, this water fog technology can stabilize and isolate the agent (traps and deposits it on the ground, avoiding its expansion) without using dangerous materials or methodologies (only using water and air). Moreover, it can be independently activated as soon as the potential attack is known if people are present in the area. This allows for a decrease in the agent load in the air so that exposure to or inhalation of the agent can be minimized. Additionally, it can be used in the decontamination phase when a disinfectant is added to create fog. 

## 5. Conclusions

Counterfog^®^ is a system built for the fast decontamination of air and the disinfection of surfaces. In this study, *Bacillus thuringiensis* spores were released into the air to assess the cleaning effectiveness of Counterfog^®^. Generally, spores remain in the air for longer than 60 min with a rate of biological removal of −0.017x + 3.450. Three kinds of artificial water fog (fogs 1, 2, and 3) were studied. These three fogs were applied for 1 min with different water and air pressures. Fog 1 reduced 99.63% of the spores in 20 min (2-log_10_ reduction). Fog 2 reduced 100% of the spores in 20 min (>3-log_10_ reduction), and fog 3 (with a larger number of the finest 2.5 µm droplets) was the fastest at removing spores from the air, achieving a higher removal rate, and reducing 100% of the spores in 20 min (>3-log_10_ reduction). 

In this study, two disinfectants were separately added to the fog to eliminate *B*. *thuringiensis* spores from porous and non-porous surfaces. The disinfectants tested were 8% hydrogen peroxide (H_2_O_2_) and 2% glutaraldehyde. First, the dispersion of the disinfectant fog was studied, showing that the Counterfog^®^ system created fog that reached all the surfaces of the test room. Surface disinfection efficiency depends on the type of material to be disinfected. Decontamination was more effective for porous than for non-porous coupons in all decontamination cases. The logarithm reduction (log_10_ reduction) ranged from 2.65 to 4.80 for a porous coupon (plasterboard); differences in log_10_ reduction were due to the position and orientation of the coupon (no statistically significant differences). The hydrogen peroxide fogs exhibited a higher log_10_ reduction in all positions and orientations as compared to the glutaraldehyde fogs. The Counterfog^®^ system was compared with a commercially available conventional fogging device. The fog generated by this device was also homogeneous, reaching a distance suitable for the decontamination of all coupons. However, in the same test conditions, the Counterfog^®^ system more efficiently reduced the number of spores in the coupons than the conventional fogging device. An important aspect of the Counterfog technology is that no transport of viable spores was observed due to the action of the jet of fog.

## Figures and Tables

**Table 1:** Number and size of particles generated by the different fogs created with Counterfog^®^.

	2.5 µm Droplets	5 µm Droplets	10 µm Droplets
Fog 1	50,000	750,000	700,000
Fog 2	50,000	750,000	350,000
Fog 3	700,000	750,000	150,000

**Table 2:** Sampling time after spore release and after fog production.

Time Since Spore Release	Time Since Water Fog Production	
0		Spore release
1 min		Sample t_1_
2 min	0	Fog production
3 min	1 min	Sample t_2_
7 min	5 min	Sample t_3_
10 min	8 min	Sample t_4_
15 min	13 min	Sample t_5_
20 min	18 min	Sample t_6_

**Table 3:** Decontamination parameters for Fog Dynamics Laboratory.

Phase	Parameter
Conditioning	Duration: 10 min Temperature: 20 °C Relative humidity: 80%
Decontamination (Fog Release)	Duration: 1 min Flow rate: 0.005 m^3^/min Temperature: 20 °C Relative humidity: 80–100%
Dwell	Duration: 60 min Temperature: 20 °C Relative humidity: 80–100%
Aeration	Duration: 10 min Flow rate: 200 m^3^/h Temperature: 20 °C Relative humidity: 70–100%

**Table 4:** Sanitization efficacy of three water fogs generated for 1 min against aerosolized *B. thuringiensis* spores. Values are expressed as logarithmic reduction and percentage reduction obtained from triplicates of three different experiments.

Samples	Fog 1		Fog 2		Fog 3	
	Log_10_ Red	% Red	Log_10_ Red	% Red	Log_10_ Red	% Red
*t*_1_	NA	NA	NA	NA	NA	NA
*t*_2_	1.26	94.58	1.66	97.83	1.90	98.75
*t*_3_	1.33	95.30	1.66	97.83	2.08	99.16
*t*_4_	0.41	61.37	0.24	42.41	2.38	99.58
*t*_5_	>3	100	2.03	99.07	2.20	99.37
*t*_6_	2.44	99.63	>3	100	>3	100

NA: not applicable.

**Table 5:** Average spore extraction efficiency of *Bacillus thuringiensis* spores from the different types of coupons.

Test Material	Mean % Recovery
Wood	12.54
Plasterboard	12.12
Steel metal	17.85
Galvanized steel	14.56
PVC sign	75.12
PVC pipe	53.39
Glass	80.53

**Table 6:** Decontamination efficacy of Counterfog^®^ jets of fog against *Bacillus thuringiensis* spores spread on coupons depending on their localization and orientation.

8% H_2_O_2_ Counterfog^®^			
Position	CFU/mL	Log10 Reduction	% Reduction
Control	2.11 × 10^8^ ± 1.73 × 10^3^	NA	NA
1	1.23 × 10^5^ ± 3.22 × 10^3^	3.24	99.94
2	3.23 × 10^5^ ± 3.16 × 10^5^	2.59	99.74
3	2.85 × 10^3^ ± 4.95 × 10^2^	4.87	99.99
4	2.01 × 10^4^ ± 1.34 × 10^4^	4.02	99.99
5	6.20 × 10^3^ ± 1.70 × 10^3^	4.53	99.99
6	5.00 × 10^4^ ± 5.52 × 10^4^	3.63	99.98
7	2.48 × 10^5^ ± 1.43 × 10^5^	2.93	99.88
8	4.00 × 10^5^ ± 7.07 × 10^4^	2.72	99.81
9	6.00 × 10^5^ ± 4.38 × 10^5^	2.55	99.72
10	3.60 × 10^4^ ± 8.49 × 10^3^	3.77	99.98
**2% Glutaraldehyde Counterfog^®^**			
**Position**	**CFU/mL**	**Log10 Reduction**	**% Reduction**
Control	6.5 × 10^7^ ± 1.84 × 10^7^	NA	NA
1	4.80 × 10^5^ ± 1.41 × 10^4^	2.13	99.26
2	5.25 × 10^5^ ± 2.90 × 10^5^	2.09	99.19
3	2.32 × 10^7^ ± 9.69 × 10^6^	0.45	64.38
4	6.05 × 10^5^ ± 8.28 × 10^5^	2.03	99.07
5	9.75 × 10^4^ ± 2.12 × 10^3^	2.82	99.85
6	4.15 × 10^6^ ± 1.77 × 10^6^	1.19	93.62
7	3.56 × 10^5^ ± 3.46 × 10^5^	2.26	99.45
8	3.10 × 10^6^ ± 7.77 × 10^5^	1.32	95.23
9	6.95 × 10^7^ ± 6.36 × 10^6^	0.02	4.62
10	4.55 × 10^3^ ± 2.19 × 10^3^	4.15	99.99

NA, not applicable.

**Table 7:** Decontamination efficacy of 8% hydrogen peroxide and 2% glutaraldehyde Counterfog^®^ against *Bacillus thuringiensis* spores spread on coupons.

8% H_2_O_2_ Counterfog^®^			
Test Material	CFU/mL	Log10 Reduction	% Reduction
Wood			
Control	3.21 × 10^6^ ± 3.53 × 10^6^	NA	NA
Decontaminated	3.50 × 10^2^ ± 4.95 × 10^2^	3.96	99.99
Plasterboard			
Control	3.23 × 10^6^ ± 2.59 × 10^4^	NA	NA
Decontaminated	2.17 × 10^2^ ± 1.65 × 10^2^	4.17	99.99
Steel metal			
Control	5.25 × 10^6^ ± 1.37 × 10^5^	NA	NA
Decontaminated	4.85 × 10^4^ ± 4.48 × 10^3^	2.03	99.08
Galvanized steel			
Control	4.83 × 10^5^ ± 3.77 × 10^4^	NA	NA
Decontaminated	8.82 × 10^3^ ± 1.01 × 10^3^	1.74	98.18
PVC sign			
Control	3.03 × 10^6^ ± 3.64 × 10^6^	NA	NA
Decontaminated	8.62 × 10^3^ ± 1.25 × 10^3^	2.54	99.72
PVC pipe			
Control	8.03 × 10^6^ ± 6.60 × 10^5^	NA	NA
Decontaminated	1.12 × 10^4^ ± 7.54 × 10^2^	2.86	99.86
Glass			
Control	1.51 × 10^6^ ± 1.41 × 10^5^	NA	NA
Decontaminated	3.17 × 10^2^ ± 2.36 × 10^1^	3.68	99.98
**2% Glutaraldehyde Counterfog^®^**			
**Test Material**	**CFU/mL**	**Log10 Reduction**	**% Reduction**
Wood			
Control	4.75 × 10^6^ ± 1.34 × 10^6^	NA	NA
Decontaminated	6.15 × 10^5^ ± 1.63 × 10^5^	0.89	87.05
Plasterboard			
Control	5.32 × 10^6^ ± 2.09 × 10^6^	NA	NA
Decontaminated	5.07 × 10^4^ ± 6.60 × 10^3^	2.02	99.05
Steel metal			
Control	5.40 × 10^6^ ± 7.07 × 10^5^	NA	NA
Decontaminated	3.69 × 10^6^ ± 8.98 × 10^5^	0.16	31.76
Galvanized steel			
Control	8.25 × 10^6^ ± 4.22 × 10^6^	NA	NA
Decontaminated	5.67 × 10^6^ ± 1.13 × 10^6^	0.16	31.31
PVC sign			
Control	4.88 × 10^6^ ± 2.80 × 10^6^	NA	NA
Decontaminated	3.31 × 10^6^ ± 1.90 × 10^6^	0.17	32.22
PVC pipe			
Control	1.38 × 10^7^ ± 1.31 × 10^7^	NA	NA
Decontaminated	4.63 × 10^6^ ± 5.94 × 10^6^	0.48	66.49
Glass			
Control	3.38 × 10^7^ ± 3.98 × 10^7^	NA	NA
Decontaminated	3.36 × 10^6^ ± 3.76 × 10^6^	1.00	90.05

NA, not applicable.

**Table 8:** Decontamination efficacy of *Bacillus thuringiensis* spores following hydrogen peroxide fog created with a commercial device.

Test Material	CFU/mL	Log10 Reduction	% Reduction
Wood			
Control	2.27 × 10^6^ ± 1.80 × 10^6^	NA	NA
Decontaminated	1.00 × 10^5^ ± 2.05 × 10^4^	1.35	95.59
Plasterboard			
Control	2.27 × 10^6^ ± 1.44 × 10^6^	NA	NA
Decontaminated	2.04 × 10^4^ ± 8.74 × 10^3^	2.05	99.10
Steel metal			
Control	2.47 × 10^6^ ± 9.57 × 10^5^	NA	NA
Decontaminated	5.44 × 10^5^ ± 2.73 × 10^5^	0.66	77.98
Galvanized steel			
Control	1.97 × 10^6^ ± 2.97 × 10^5^	NA	NA
Decontaminated	1.00 × 10^6^ ± 3.70 × 10^5^	0.29	49.07
PVC sign			
Control	4.73 × 10^7^ ± 5.84 × 10^7^	NA	NA
Decontaminated	3.15 × 10^6^ ± 9.88 × 10^5^	1.18	93.35
PVC pipe			
Control	1.74 × 10^7^ ± 3.66 × 10^6^	NA	NA
Decontaminated	3.02 × 10^6^ ± 1.78 × 10^6^	0.76	82.62
Glass			
Control	2.38 × 10^7^ ± 1.53 × 10^7^	NA	NA
Decontaminated	5.72 × 10^5^ ± 5.40 × 10^5^	1.62	97.60

NA, not applicable.

## Data Availability

The data presented in this study are available in the present article.
